# Perinatal depression among mothers in a South African birth cohort study: Trajectories from pregnancy to 18 months postpartum

**DOI:** 10.1016/j.jad.2019.08.052

**Published:** 2019-12-01

**Authors:** Jennifer A. Pellowski, Angela M. Bengtson, Whitney Barnett, Kira DiClemente, Nastassja Koen, Heather J. Zar, Dan J. Stein

**Affiliations:** a Department of Behavioral and Social Sciences, International Health Institute, Brown University School of Public Health, Providence, RI, USA; b Department of Epidemiology, Brown University School of Public Health, Providence, RI, USA; c Department of Paediatrics and Child Health, Red Cross War Memorial Children's Hospital and South African Medical Research Council (SAMRC) Unit on Child & Adolescent Health, University of Cape Town, South Africa; d Department of Psychiatry and Mental Health and South African Medical Research Council (SAMRC) Unit on Risk & Resilience in Mental Disorders, Neuroscience Institute, University of Cape Town, South Africa

**Keywords:** Perinatal depression, Group-based trajectory model, South Africa

## Abstract

**Background:**

Perinatal depression affects 21–50% of women in South Africa and poses significant health risks to mothers and children. Trajectories of depressive symptoms change over time and have not been well characterized during the perinatal period in low and middle-income countries.

**Methods:**

Data from women enrolled in a population-based birth cohort study in Paarl, South Africa with at least 3 depression measures from pregnancy through 18 months postpartum (*N* = 831) were analyzed. Depressive symptoms were measured continuously using the Edinburgh Postnatal Depression Scale (EPDS). Group-based trajectory models were used to estimate trajectories of depressive symptoms during the perinatal period and multinomial multivariable models to identify predictors of trajectory group membership.

**Results:**

Five distinct trajectory patterns of depressive symptoms were identified: moderate levels of depressive symptoms during pregnancy but minimal postpartum (3.5%), minimal levels during pregnancy and increasing postpartum (3.7%), unstable levels peaking at 12 months postpartum (6.6%), mild levels with slight decrease postpartum (82.9%), and severe levels during pregnancy and postpartum (3.1%). Membership in the chronic severe symptom group was associated with stressful life events, sexual intimate partner violence and tobacco use.

**Limitations:**

Modeling limitations prevented determining how changes in psychosocial predictors over time may influence depressive symptom trajectories.

**Conclusions:**

Mild to severe depressive symptoms during pregnancy/postpartum were common among this South African cohort. Interventions to treat women with severe chronic depressive symptoms with co-occurring psychosocial issues are urgently needed.

## Highlights

• Perinatal depressive symptom trajectories are understudied in sub-Saharan Africa.
• A majority of women had mild levels of depressive symptoms.
• The group with persistent severe symptoms had the most risk factors.
• Depressive symptom trajectories differed by community context.

## 1 Introduction

Perinatal depression is common globally, with approximately 11.9% of women experiencing perinatal depression, with the highest burden (19.2% during pregnancy; 18.7% postpartum) in low and middle-income countries (LMIC; Woody et al., 2017). Maternal depression during pregnancy and the postpartum period has been linked to a number of adverse child health outcomes and maternal-child relationship factors (Field, 2011, Kingston et al., 2012). Depression during the pregnant and postpartum period has been associated with reduced infant cognitive development (Stein et al., 2014), socio-emotional development (Feldman et al., 2009, Leis et al., 2014), and psychomotor development (Field et al., 2008). These influences have been found in high, middle, and low-income countries (Gelaye et al., 2016, Stein et al., 2014). In terms of maternal health, longer durations of untreated depression are associated with poorer clinical outcomes for mother and child, such as longer response times to treatment and relapse, as well as higher rates of depression-related disability (Ghio et al., 2015, Jarde et al., 2016).

In low and middle-income countries, rates of perinatal depression are elevated compared to global levels (WHO, 2018). Previous studies conducted in South Africa have found antenatal depression rates between 21 and 47% (Brittain et al., 2015, Manikkam and Burns, 2012, Rochat et al., 2011, van Heyningen et al., 2016) and postpartum rates between 31.7% and 50.3%, much higher than global perinatal depression rates (Dewing et al., 2013, Peltzer et al., 2018 Stellenberg and Abrahams, 2015). Estimates of depression rates within South Africa may differ for a number of reasons including measurement type and related validity (e.g. self-report symptom scale versus clinician assessment), timing of measurement (e.g. measurements during pregnancy versus early postpartum (days/weeks) versus up to 18 months postpartum) and sample population (e.g. general population-based cohorts versus special populations, such as women living with HIV).

Regardless due to the high prevalence of perinatal depression, especially given the low resource environment, it is imperative to be able to target perinatal depression prevention and intervention efforts in LMICs towards those most likely at risk. For women living in LMICs, outside of pregnancy there is often limited engagement with health services, including mental health referrals, further underlining the importance of understanding depressive symptom trajectories and predictors of those trajectories during pregnancy. Moreover, women living in LMICs often have the additional burden of comorbidities, such as HIV, and social contextual factors, such as high levels of unemployment, food insecurity and exposure to trauma or violence, that may exacerbate depressive symptoms or change patterns of symptoms across time. Risk for depression could present during pregnancy but may also appear any time postpartum. (Banti et al., 2011)

Given the longitudinal nature of perinatal depression, understanding patterns of depression across the transition from pregnancy to postpartum and throughout the postpartum period is paramount. However, merely assessing the average longitudinal trajectory across a cohort masks cohort heterogeneity as well as important nuances related to timing, chronicity, and severity (Garman et al., 2019, Santos et al., 2017). Depressive symptoms tend to be cyclical and episodic, making it important to understand the trajectory of patient's depressive symptoms over time. Group-based trajectory models allow researchers to capture the underlying heterogeneity in perinatal depression symptoms present in cohorts, to determine risk profiles and identify chronic versus transient patterns as well as determine, for those with transient patterns, whether symptoms decrease over time or increase.

A recent review of studies on perinatal depression found 11 studies that use growth curve mixture modeling (Baron et al., 2017). Of these studies, all consistently found a stable low risk trajectory and most found a stable moderate-high or high risk of depression trajectory. About half reported a transient trajectory in which depressive symptoms were either increasing, decreasing, or episodic. This review also noted several limitations including that none of these studies were conducted in a low or middle-income country. Given the elevated prevalence rates in LMICs, studies are needed to determine perinatal depression trajectories in these settings.

Effective strategies for reducing symptoms of postpartum depression exist (Surkan et al., 2016), however, to optimize limited mental health resources in LMICs, it is essential to identify women at high risk for developing perinatal depression over time through determining antenatal predictors of these specific perinatal depression trajectories. Previous studies conducted in high-income settings have not found consisted predictors of depression trajectory types (Baron et al., 2017), however, commonly tested variables include education, income, social support, exposure to stress, anxiety, alcohol and tobacco use, and history of abuse. Given differences in levels of exposure to many of these variables in LMICs versus high-income countries, it is imperative to test these associations in resource-limited settings.

The purpose of this study was to determine the patterns of perinatal depressive symptoms among a cohort of pregnant women living in South Africa for whom perinatal depression data was collected through 18 months postpartum. We also sought to characterize longitudinal trajectories of depressive symptoms from pregnancy to 18 months postpartum and identify predictors of those trajectories to determine modifiable or co-morbid risk factors. This study provides novel insight into depressive symptom trajectories of perinatal women in LMIC, which is needed to identify those at-risk for depression during this high-risk period and to prevent the negative health outcomes for mothers and children.

## 2 Methods

The Drakenstein Child Health Study (DCHS) is a population-based birth cohort study conducted in two low socio-economic, peri‑urban communities in Paarl, South Africa (Zar et al., 2015). Between March 2012 and March 2015 the cohort enrolled 1225 pregnant women during their second trimester and followed the mother-child dyads up to 5 years postpartum. Enrolment occurred at 2 public sector clinics – TC Newman, serving a mixed ancestry community and Mbekweni serving a Black South African community. These two communities have marked cultural, linguistic, and historical differences but both communities represent key populations for further understanding maternal and child health in a resource-restricted context. In this cohort, biomedical, environmental, psychosocial and demographic risk factors for poor child health were collected longitudinally. The current analyses utilize the maternal psychosocial data available at pregnancy and up to 18 months postpartum (Stein et al., 2015). Ethics approval for DCHS was obtained through the Human Research Ethics Committee (HREC) of the Faculty of Health Sciences, University of Cape Town, Stellenbosch University, and the Western Cape Provincial Research committee. All participants completed informed consent (Stein et al., 2015, Zar et al., 2015)

### 2.1 Perinatal depressive symptoms

The Edinburgh Postnatal Depression Scale (EPDS) was used to assess symptoms of depression in the past 7 days at five time points during pregnancy and postpartum: the second trimester of pregnancy, and 10 weeks, 6 months, 12 months, and 18 months postpartum. The EPDS has been validated in both postpartum and pregnant women (Cox et al., 1987, Murray and Cox,). The scale was completed in participants’ home language or language of choice (English, isiXhosa, or Afrikaans). The EPDS has previously been validated in all three languages (de Bruin et al., 2004, Lawrie et al., 1998, Murray and Cox, van der Westhuizen et al., 2018). This self-report measure consists of 10 items that are rated on a scale of 0 to 3. The scores are summed for a maximum value of 30. In this analysis, depressive symptoms were measured as a participant's continuous EPDS score over time. In this sample, the EPDS exhibited acceptable internal reliability (Cronbach's alpha=0.78). We characterized the levels in severity of symptoms as follows: scores of 0–6 indicating none or minimal depressive symptoms, 7–13 indicating mild depressive symptoms, 14–18 indicating moderate depressive symptoms, and 19–30 indicating severe depressive symptoms (McCabe-Beane et al., 2016).

### 2.2 Demographic and psychosocial predictors

We assessed whether baseline demographic and psychosocial variables during pregnancy correlated with particular maternal perinatal depressive symptom trajectories. Socioeconomic status (SES) was calculated using current employment, standardized scores of educational attainment and household income and a composite asset index (Myer et al., 2008, Brittain et al., 2015). SES is reported by quartiles and participants are categorized as low, low-moderate, moderate-high, and high SES compared to other participants in the cohort. Participants were asked whether or not they received social assistance grants, which are common sources of household financial resources in South Africa. Marital status and whether or not the pregnancy was planned was collected at baseline. HIV status was determined through HIV testing during pregnancy. The term ‘community’ is used to indicate the clinic from which participants were recruited (i.e. TC Newman and Mbekweni). Between these two communities there are very low levels of clinic cross-over, meaning if a woman is from Mbekweni community she is very unlikely to go to the TC Newman clinic.

Stressful life events during the past 12 months were reported using the World Mental Health Life Events Questionnaire (Myer et al., 2008). Individual items are scored according to whether or not the participant experienced the events (0=no, 1=yes) with a maximum total score of 17, with higher scores indicating greater exposure to life events. Childhood trauma was assessed using the Childhood Trauma Questionnaire – Short Form (CTQ-SF; Bernstein et al., 1994). Each item is assessed on a Likert scale ranging from 1 = *never true* to 5 = *very often true*. For the purposes of these analyses, the total score from all subscales was used and utilizing established guidelines participants were categorized as above (Total Score >36) or below (Total Score ≤ 36) the threshold of experiencing childhood trauma (Bernstein and Fink, 1998).

Recent experiences of intimate partner violence (IPV) was self-reported using an adapted measure from the WHO multi-country study (Jewkes, et al., 2002) and the Women's Health Study in Zimbabwe (Shamu et al., 2011). Each item is assessed on a Likert scale ranging from 1 = *never* to 4 = *many times*. Participants were coded as having experienced recent (within the last 12 months) emotional IPV if they had a score of 6 or greater on the emotional IPV subscale, experienced physical IPV if they had a score of 7 or greater on the physical IPV subscale, and experienced sexual IPV if they had a score of 5 or greater on the sexual IPV subscale (Dunkle et al., 2004). Smoking tobacco and drinking alcohol during pregnancy was self-reported using the ASSIST (WHO ASSIST Working Group, 2002). Tobacco use and alcohol use were scored as *any use during pregnancy* and *no use during pregnancy* (World Health Organization, 2010).

### 2.3 Statistical analyses

The sample utilized for these analyses consisted of participants who had data from 3 or more of the time points specified (pregnancy, 10 weeks, 6 months, 12 months, and 18 months postpartum). Group-based trajectory models can handle missing data using maximum likelihood estimation, however, they can become very unstable with high levels of missing data. Restricting the dataset to participants who had data from 3 or more time points balanced managing the amount of missing data to reduce instability while maximizing the number of participants included in these analyses. Comparison analyses were conducted using t-tests and chi squares between those who were included in these analyses and those who were excluded to determine if there were significant differences in baseline depressive symptoms or baseline demographic or psychosocial predictor variables.

Baseline demographic and psychosocial variables are reported in N's and percentages by community. Group-based trajectory models were estimated using PROC TRAJ using SAS 9.4 (Jones and Nagin, 2007). Group-based trajectory modeling estimates trajectories of depressive symptoms by grouping participants together with similar patterns of depressive symptoms over time. The continuous total score on the EPDS was used to model depressive symptoms trajectories, therefore, increases or decreases in depressive symptom trajectory correspond to a one-unit change in EPDS score. Group based trajectory models were specified using the censored normal distribution to account for the minimum (0) and maximum values (30) of the EPDS scale. Additionally, we specified a random intercept to account for intra-participant correlation in depressive symptoms over time.

Optimal trajectory groups were determined using a two-step process. First, the optimal number of trajectory groups in the study population was determined using the Bayesian Information Criterion (BIC value) from models allowing for a differing numbers of trajectory groups. To maintain interpretability and clinical relevance, the maximum number of groups was capped at 6 groups. In this step, all trajectory groups were modeled using cubic polynomial terms. Second, after the number of trajectory groups was selected, the optimal functional form (e.g. linear, quadratic, cubic) for each trajectory group was determined using the BIC and graphical inspection of the data. Graphical inspection was used first to determine plausible functional forms for each trajectory. BICs were used to compare model fit between the original model and any changes in functional forms. Changes in functional forms were done for one group at a time to ensure proper precision in BIC comparisons. Group based trajectory models assign each subject a posterior probability for each group. The posterior probability measures an individual probability of belonging to a particular group based on her measured depressive symptom scores across time. Adequacy of the final group-based trajectory model was assessed by (1) examining the correspondence between the estimated probability of group membership and the proportion of participants assigned to each group based on the model-estimated posterior probability of group membership and (2) assessing if the median posterior probability of group membership was ≥ 0.70 for all groups. To test the assumption of data missing completely at random (MCAR), Little's MCAR was used as well as its extension – covariate-dependent missingness (CDM) to determine if missingness was dependent upon group trajectory using Stata and the mcartest command (Li, 2013, Little, 1988).

The full group-based trajectory model presented does not include predictors because of instability in the model when trying to enter predictors. Instead, to investigate baseline (pregnancy) predictors of group membership, a multinomial multivariate regression model was used. For the outcome of group membership, each participant was assigned a group based on the group with the maximum posterior probability of group membership. In this multinomial multivariate regression model, the group with the largest proportion of the sample is used as the referent category; thus, all predictor findings for specific groups are described in relation to this referent category group. Possible predictors were identified from the literature and tested using bivariate multinomial regressions. These predictors included: community, SES (used as a continuous measure to aid in interpretability), receipt of social grant, marital status, planned pregnancy, child trauma, stressful life events, intimate partner violence (emotional, physical and sexual), tobacco use during pregnancy, alcohol use during pregnancy, and HIV status. Predictors for which *p*<0.10 for at least one group in bivariate analyses were entered into the multivariate multinomial regression model. Adjusted odds ratios (AOR) are reported and all variables included in the model are displayed.

## 3 Results

Of the 1225 participants enrolled in the DCHS cohort, 831 completed three or more of the EPDS assessments and were included in these analyses. Participants who completed three or more EPDS assessments were significantly different from those who completed 2 or fewer assessments on several baseline (pregnancy) demographic and psychosocial variables; participants who completed three or more EPDS assessments were more likely to be from TC Newman, more likely to have a lower socioeconomic status, more likely to have received a social assistance grant, and more likely to have used any tobacco during pregnancy. Participants who were included in these analyses were not significantly different on any other demographic or psychosocial variables. Baseline (pregnancy) depression score was not associated with completing three or more EPDS assessments.

Table 1 shows maternal demographic and psychosocial characteristics collected at baseline by community. Only 36.5% (*n* = 303) of the sample had completed secondary education and about a quarter were employed during pregnancy (25.8%, *n* = 213). The majority of women had a household income of R1000-R5000 per month (equivalent of $74 - 382/month), and half received social assistance grants (49.8%, *n* = 412). At baseline, 39.3% were married or cohabitating, and 66.5% of the pregnancies were unplanned. For psychosocial characteristics, the mean number of stressful life events experienced in the past year was 1.9, 34.7% of the sample met criteria for experiencing childhood trauma, and 34.5% experienced any type of intimate partner violence in the past year. The rate of any self reported smoking during pregnancy was 29.4% and 16.4% of participants reported consuming alcohol while pregnant. Many of these demographic and psychosocial characteristics significantly varied by community (Table 1).

**Table 1:** Maternal demographic and psychosocial characteristics during pregnancy by community.

	Total		TC Newman (Mixed ancestry)		Mbekweni (Black African)		
	n	(%)	n	(%)	n	(%)	X^2^
Number of mothers	831	(100)	394	(47.4)	437	(52.6)	
Completed secondary education	303	(36.5)	152	(38.7)	151	(34.6)	1.52
Employed (full or part-time)	213	(25.8)	111	(28.4)	102	(23.4)	2.63
Average household income							
<R1,000/mo	314	(38.7)	116	(30.8)	198	(45.5)	35.87⁎⁎⁎
R1,000-R5,000/mo	408	(50.2)	200	(53.1)	208	(47.8)	
R5,000-R10,000/mo	76	(9.4)	47	(12.5)	29	(6.7)	
R10,000-R15,000/mo	9	(1.1)	9	(2.4)	0	(0)	
>R15,000/mo	5	(0.6)	5	(1.3)	0	(0)	
SES quartile							
Lowest SES	216	(26)	71	(18)	145	(33.2)	31.17⁎⁎⁎
Low-moderate SES	225	(27.1)	109	(27.7)	116	(26.5)	
Moderate-high SES	213	(25.6)	107	(27.2)	106	(24.3)	
Highest SES	177	(21.3)	107	(27.2)	70	(16)	
Receives social grants	412	(49.8)	191	(48.8)	221	(50.6)	0.25
Married/cohabiting	326	(39.3)	173	(44)	153	(35.1)	6.91⁎⁎
Unplanned pregnancy	518	(66.5)	234	(62.6)	284	(70.1)	4.98*
HIV positive	185	(22.3)	14	(3.6)	171	(39.1)	151.54⁎⁎⁎
Any antenatal smoking	229	(29.4)	205	(55)	24	(5.9)	224.77⁎⁎⁎
Any antenatal alcohol use	127	(16.4)	97	(25.9)	30	(7.5)	48.10⁎⁎⁎
Childhood trauma (above threshold)	271	(34.7)	163	(43.6)	108	(26.6)	24.76⁎⁎⁎
Recent IPV (any type)	269	(34.5)	154	(41.2)	115	(28.4)	14.05⁎⁎⁎
Emotional IPV	217	(27.9)	134	(35.8)	83	(20.5)	22.75⁎⁎⁎
Physical IPV	179	(23)	96	(25.7)	83	(20.5)	2.94
Sexual IPV	55	(7.1)	41	(11)	14	(3.5)	16.69⁎⁎⁎
	M	SD	M	SD	M	SD	*t*-test
Stressful life events	1.9	2.2	2.6	2.4	1.4	1.9	8.17⁎⁎⁎
Depression (EPDS)							
Antenatal	9.5	5.2	9.1	6.1	9.9	4.2	−2.31*
10 weeks postpartum	8.1	5	7.1	5.9	9	3.8	−4.79⁎⁎⁎
6 months postpartum	7.9	5.1	7.6	6.2	8.2	4	−1.49
12 months postpartum	7.7	5.6	7.6	6.5	8	4.5	−0.88
18 months postpartum	5.7	5.3	5.6	6.2	5.7	4.2	−0.15

Note:.

⁎ =*p*<0.05.

⁎⁎ =*p*<0.01.

⁎⁎⁎ =*p*<0.001.

### 3.1 Proportion of women with depressive symptoms

Among the full sample (Table 2), during the second trimester of pregnancy, 24.2% (*n* = 189) of the sample met criteria for probable depression on the Edinburgh Postnatal Depression Scale (EPDS). Overall, depressive symptoms decreased postpartum; at 10 weeks postpartum 17.1% (*n* = 106) had probable depression, at 6 months postpartum 14.5% (*n* = 87) had probable depression, 12 months postpartum 16.5% (*n* = 110) had probable depression, and at 18 months postpartum 10.2% (*n* = 60) had probable depression. During pregnancy, the median depressive symptom score was 9 [IQR: 6, 12], which decreased to 8 [IQR: 5, 11] at both 10 weeks and 6 months postpartum. This downward trend continued later postpartum with a median score of 7 [IQR: 3.5, 11] at 12 months postpartum and a median score of 6 [IQR: 0,8] at 18 months postpartum.

**Table 2:** Depressive symptoms as measured by the Edinburgh Postnatal Depression Scale (EPDS) during pregnancy at 10 weeks, 6 months, 12 months, and 18 months postpartum.

	Second trimester of pregnancy		10 weeks postpartum		6 months postpartum		12 months postpartum		18 months postpartum	
	n	%	n	%	n	%	n	%	n	%
Number of participants meeting criteria for depression^	189	24.2%	106	17.1%	87	14.5%	110	16.5%	60	10.2%
	Median depression score	IQR	Median depression score	IQR	Median depression score	IQR	Median depression score	IQR	Median depression score	IQR
Total Sample	9	[6, 12]	8	[5, 11]	8	[5, 11]	7	[3.5, 11]	6	[0, 8]
Missing Data N,%	51	6.1%	212	25.5%	229	27.6%	163	19.6%	242	29.1%
Group 1 - 82.9% of sample+	9	[6, 12]	8	[5, 11]	8	[8, 10]	7	[3, 9]	6	[1, 8]
Missing Data N (%)	50	6.8%	184	25%	199	27%	145	19.7%	228	30.9%
Group 2 - 3.7% of sample	5.5	[2.75, 9.25]	7	[3, 9]	11.5	[7, 15.25]	13.5	[10.5, 21]	16	[14, 20]
Missing Data N (%)	0	0%	3	16.7%	6	33.3%	4	22.2%	1	5.6%
Group 3 - 6.6% of sample	11	[7.5, 13.5]	3	[0, 6.5]	9	[6, 16]	20	[17, 21]	0	[0, 0]
Missing Data N (%)	1	2.2%	13	28.3%	17	37%	9	19.6%	9	19.6%
Group 4 - 3.5% of sample	17	[14, 20]	8	[2.5, 20]	1	[0, 4.5]	0	[0, 1]	0	[0, 0]
Missing Data N (%)	0	0%	2	18.2%	2	18.2%	3	27.3%	1	9.1%
Group 5 - 3.1% of sample	19	[17, 23]	21	[18, 24]	19	[12.5, 24]	19	[16.5, 21]	17.5	[16, 21.5]
Missing Data N (%)	0	0%	10	52.6%	5	26.3%	2	10.5%	3	15.8%

Notes:.

^ percentages out of available data for each time point; +Percentages of the sample for each group are based on latent trajectory model. Data in the table is based on model estimated posterior probabilities of group membership.

### 3.2 Depression trajectory model and description of groups

**Fig. 1:**
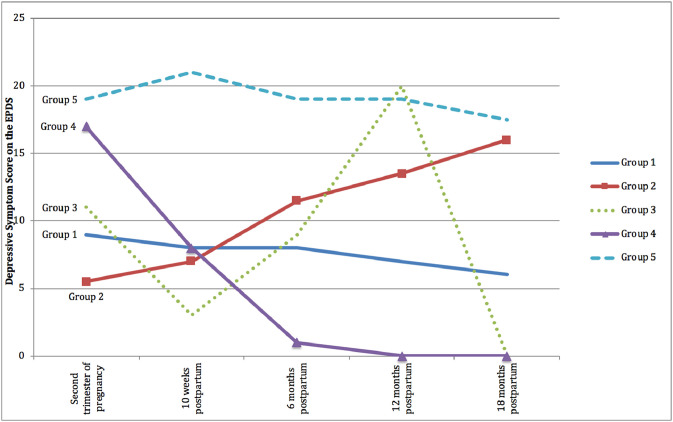
Depressive symptom trajectories for 831 pregnant women through 18 months postpartum. Depressive symptoms were measured using the Edinburgh Postnatal Depression Scale (EPDS). Group 1 (Mild, slight decrease postpartum – 82.9%) was modeled using a linear term, Group 2 (Minimal during pregnancy, increasing postpartum – 3.7%) was modeled using a cubic term, Group 3 (unstable, peak at 12 months postpartum – 6.6%) was modeled using a cubic term, Group 4 (Moderate during pregnancy, minimal postpartum – 3.5%) was modeled using a linear term, and Group 5 (Severe during pregnancy and postpartum – 3.1%) was modeled using a linear term.

The model with 5 trajectory groups fit the best (BIC=−9376.48, AIC=−9315.60, LogLikelihood= −9295.60; Fig. 1, Table 2). The optimal functional forms included three linear group trajectories and two cubic group trajectories. Percentages presented here are from the group-based trajectory model estimates. Group 1 (“Mild during pregnancy, slight decrease postpartum”, modeled using a linear term) consisted of the majority of the sample (82.9%), which had mild levels of depressive symptoms during pregnancy that did not meet the EPDS threshold for probable depression. These depressive symptoms decrease slightly postpartum but were still elevated. Group 2 (“Minimal during pregnancy, increasing postpartum”, modeled using cubic term) consisted of 3.7% of the sample and started with low levels of depressive symptoms during pregnancy, which increased postpartum and were above the threshold for probable depression by 12 and 18 months postpartum. Group 3 (“Unstable, peak at 12 months postpartum”, modeled using a cubic term) was comprised of 6.6% of the sample. In this group, participants had mild, but below threshold, levels of depressive symptoms during pregnancy which decreased early postpartum (10 weeks). Later postpartum these symptoms increase peaking at 12 months postpartum with very severe levels of depressive symptoms, and then dissipated to little to no depressive symptoms by 18 months postpartum. Group 4 (“Moderate during pregnancy, minimal postpartum”, modeled using a linear term) consisted of 3.5% of the sample and is characterized by moderate levels of depressive symptoms during pregnancy that decrease substantially postpartum. Group 5 (“Severe during pregnancy and postpartum”, modeled using a linear term) consisted of 3.1% of the sample and was characterized by persistent high levels of depressive symptoms that meet the EPDS threshold for probable depression and these symptoms did not dissipate over time.

Participants were assigned to a depressive symptom trajectory group based on each participant's highest predicted posterior group membership probability. These proportions were relatively close to the estimated probability of group membership (Group 1: estimated 82.9% vs. 88.7% assigned; Group 2: estimated 3.7% vs. 2.2% assigned; Group 3: estimated 6.6% vs. 5.5% assigned; Group 4: estimated 3.5% vs. 1.3% assigned; Group 5: estimated 3.1% vs. 2.3% assigned). The median posterior probability of group membership was ≥ 0.70 for all groups (Table 3).

**Table 3:** Demographic and psychosocial variables during pregnancy by assigned depressive symptom trajectory group (based on each participant's highest predicted posterior group-membership probability).

	Group 1		Group 2		Group 3		Group 4		Group 5	
	Mild during pregnancy, slight decrease postpartum		Mild during pregnancy, increasing postpartum		Unstable, Peak at 12 months postpartum		Moderate during pregnancy, minimal postpartum		Severe during pregnancy and postpartum	
	Median	IQR	Median	IQR	Median	IQR	Median	IQR	Median	IQR
Posterior probabilities by group	0.96	[0.89, 0.99]	0.71	[0.53, 0.86]	0.79	[0.58, 0.96]	0.74	[0.59, 0.93]	0.7	[0.57, 0.92]
	n	(%)	n	(%)	n	(%)	n	(%)	n	(%)
Number of mothers	737	(88.7)	18	(2.2)	46	(5.5)	11	(1.3)	19	(2.3)
Community										
TC Newman	325	(44.1)	10	(55.6)	31	(67.4)	9	(81.8)	19	(100)
Mbekweni	412	(55.9)	8	(44.4)	15	(32.6)	2	(18.2)	0	(0)
Completed secondary education	274	(37.2)	6	(33.3)	16	(34.8)	5	(45.5)	2	(10.5)
Employed (full or part-time)	195	(26.6)	3	(17.6)	8	(17.4)	3	(27.3)	4	(21.1)
Average household income <R1,000/mo	285	(38.7)	8	(44.4)	12	(26.1)	4	(36.4)	5	(26.3)
SES quartile										
Lowest SES	193	(26.2)	5	(27.8)	10	(21.7)	1	(9.1)	7	(36.8)
Low-moderate SES	201	(27.3)	4	(22.2)	10	(21.7)	3	(27.3)	7	(36.8)
Moderate-high SES	182	(24.7)	5	(27.8)	19	(41.3)	5	(45.5)	2	(10.5)
Highest SES	161	(21.8)	4	(22.2)	7	(15.2)	2	(18.2)	3	(15.8)
Receives social grants	365	(49.7)	5	(29.4)	29	(63)	5	(45.5)	8	(42.1)
Married/cohabiting	289	(39.3)	5	(27.8)	21	(45.7)	3	(27.3)	8	(42.1)
Unplanned pregnancy	234	(34.1)	6	(33.3)	10	(22.2)	3	(27.3)	8	(42.1)
HIV positive	172	(23.3)	2	(11.1)	10	(21.7)	1	(9.1)	0	(0)
Any antenatal smoking	186	(27.2)	7	(38.9)	17	(37.8)	4	(36.4)	15	(78.9)
Any antenatal alcohol use	106	(15.5)	3	(16.7)	7	(15.6)	1	(9.1)	10	(52.6)
Childhood trauma (above threshold)	224	(32.6)	8	(44.4)	20	(44.4)	4	(36.4)	15	(78.9)
Recent IPV (any type)	220	(32.1)	7	(38.9)	22	(48.9)	6	(54.5)	14	(73.7)
Emotional IPV	174	(25.4)	5	(27.8)	20	(44.4)	5	(45.5)	13	(68.4)
Physical IPV	140	(20.4)	7	(38.9)	17	(37.8)	4	(36.4)	11	(57.9)
Sexual IPV	39	(5.7)	1	(5.6)	3	(6.7)	3	(27.3)	9	(47.4)
Median Stressful life events [IQR]	1	[0, 3]	1.5	[0, 4]	1	[0, 4]	3	[3, 7]	5	[2, 7]

Little's MCAR test was non-significant indicating that the null hypothesis that data are missing completely at random could not be rejected (X^2^=43.92, *p* = 0.71). Furthermore, the Little's CDM test with conducted with group trajectory as a covariate, which was significant, indicating we should reject the null hypothesis that missingness is dependent upon the covariate group trajectory (X^2^= 130.70, *p* = 0.02).

### 3.3 Predictors of depression trajectory groups

Table 3 displays demographic and psychosocial variables during pregnancy by assigned depressive symptom trajectory group. We were unable to fit predictors within the group-based trajectory model because of instability, thus, we examined predictors of the posterior probability of group membership. Of note, no participants from Mbekweni and no HIV positive participants were in Group 5 (Severe during pregnancy and postpartum). Because of this, community and HIV status could not included as predictors in the multinomial logistic regression. Group 1, (Mild during pregnancy, slight decrease postpartum), was chosen as the referent group for the bivariate and multinomial logistic regression analyses because it was comprised of the largest portion of the sample and it was the only group in our sample that had levels of depressive symptoms below the EPDS threshold at all time points.

Table 4 displays the results of the multinomial multivariate regression model, which tests predictors of depressive symptom trajectory group membership. Participants who reported recent physical intimate partner violence while pregnant were more likely to be in Group 2 (Minimal during pregnancy, increasing postpartum) compared to Group 1 (Mild during pregnancy, slight decrease postpartum; AOR = 3.764, *p*<0.05). In the multinomial logistic regression, there were no variables that significantly predicted participants being in Group 3 (Unstable, peak at 12 months postpartum). Participants who reported more stressful life events during pregnancy were more likely to be in Group 4 (Moderate during pregnancy, minimal postpartum) compared to Group 1 (Mild during pregnancy, slight decrease postpartum; AOR = 1.346, *p* < 0.01).

**Table 4:** Multinomial multivariate regression model predicting depressive symptom trajectory group, as defined by participant's maximum posterior probability for group membership.

Group Number	Group 1	Group 2		Group 3		Group 4		Group 5	
Description of Group Trajectory	Mild during pregnancy, slight decrease postpartum	Mild during pregnancy, increasing postpartum		Unstable, Peak at 12 months postpartum		Moderate during pregnancy, minimal postpartum		Severe during pregnancy and postpartum	
Variables	(referent group)	Adj. OR	[95% CI]	Adj. OR	[95% CI]	Adj. OR	[95% CI]	Adj. OR	[95% CI]
SES	–	1.001	[0.796, 1.261]	1.005	[0.867, 1.165]	1.078	[0.797, 1.458]	0.846	[0.643, 1.112]
Receives social grant	–	0.416	[0.143, 1.211]	1.582	[0.840, 2.980]	0.592	[0.160, 2.191]	0.548	[0.189, 1.592]
Any Tobacco use during pregnancy	–	1.908	[0.645, 5.642]	1.417	[0.705, 2.848]	1.204	[0.320, 4.538]	4.351*	[1.251, 15.134]
Any Alcohol use during pregnancy	–	0.918	[0.234, 3.610]	0.642	[0.261, 1.580]	0.372	[0.043, 3.208]	2.161	[0.733, 6.375]
Childhood Trauma Above Threshold	–	1.249	[0.433, 3.600]	1.374	[0.713, 2.649]	0.564	[0.134, 2.377]	2.307	[0.655, 8.119]
Any recent Emotional IPV	–	0.569	[0.146, 2.224]	1.632	[0.738, 3.609]	1.62	[0.350, 7.493]	1.706	[0.444, 6.562]
Any recent Physical IPV	–	3.764*	[1.111, 12.752]	1.668	[0.749, 3.714]	0.868	[0.164, 4.587]	0.765	[0.187, 3.134]
Any recent Sexual IPV	–	0.67	[0.076, 5.927]	0.515	[0.141, 1.885]	3.591	[0.699, 18.456]	6.778**	[2.001, 22.960]
Stressful Life Events	–	0.96	[0.752, 1.227]	1.056	[0.925, 1.205]	1.346**	[1.081, 1.677]	1.259*	[1.049, 1.510]

*p<0.05.

**p<0.01.

***p<0.001.

Participants who reported more stressful life events during pregnancy were more likely to be in Group 5 (Severe during pregnancy and postpartum) compared to Group 1 (Mild during pregnancy, slight decrease postpartum; AOR = 1.259, *p* < 0.01). Additionally, participants were reported any tobacco use during pregnancy were more likely to be in Group 5 compared to Group 1 (AOR = 4.351, *p* < 0.05). Finally, participants who reported any recent sexual intimate partner violence were 6 times more likely to be in Group 5 compared to Group 1 (AOR=6.778, *p*<0.001).

## 4 Discussion

The purpose of this study was to characterize perinatal depressive symptom trajectories over time among a cohort of South African women of low socioeconomic status. This is one of the first studies to investigate depressive symptom trajectories among pregnant and postpartum women living in sub-Saharan Africa. Compared to global perinatal depression rates (Baron et al., 2017), women in this sample had greater depressive symptoms and more women met criteria for probable depression. Given that depression rates in low and middle-income countries are generally higher and past studies conducted in South Africa have found similar or even higher rates, our prevalence of depression was anticipated.

Similar to previous studies (see Baron et al., 2017), we found one group (Group 5) with persistently high levels of depressive symptoms throughout pregnancy and postpartum and, thus, the most in need of intervention efforts. This group also had the most risk factors associated with membership, potentially making women with this trajectory more easily identifiable compared to women in some of the other groups. Those with persistently high levels of depressive symptoms were significantly more likely to have greater exposure to recent stressful life events and to have recent experiences with sexual IPV. This finding underscores the need for further understanding and treatment of co-occurring psychosocial issues during the peripartum period. Current literature outlines strong links between depression, IPV and stressful life events (Schneider et al., 2018, Tsai et al., 2016), however, it is unclear how these psychosocial issues may or may not interact to influence depression trajectories across time, in general, and during the pregnancy-postpartum transition specifically. Furthermore, women with persistently high levels of depressive symptoms (Group 5) were also significantly more likely to report using tobacco during pregnancy. At least one other study in the literature has found an association between tobacco use and a high perinatal depression trajectory (Glasheen et al., 2013). Tobacco use, in conjunction with recent IPV, and exposure to stressful life events, as significant predictors of persistently high depressive symptoms in this study highlight the need to simultaneously target co-occurring psychosocial and tobacco use issues.

Of note, there were no participants from Mbekweni in Group 5 who had persistently high levels of depression during pregnancy and postpartum. This underscores some of the psychosocial differences between these communities, which are likely linked to community level factors. Prevalence of all subtypes of intimate partner violence, childhood trauma and stressful life events are higher in TC Newman compared to Mbekweni. These constellations of psychosocial issues may contribute to differential trajectory patterns by community. Another potential explanation is the use of different language versions of the EPDS by community. The Afrikaans version was used in TC Newman and the isiXhosa version was used in Mbekweni. Although the EPDS has been validated in both Afrikaans and isiXhosa, there may be subtle differences in language usage rather than actual symptoms that could have contributed to these differences in group trajectory.

Group 1 comprised the majority of the sample (82.9%) and the median depressive symptom score remained somewhat elevated throughout the postpartum period indicating that a proportion of women may have had mild to moderate levels of depressive symptoms throughout pregnancy and postpartum. This finding of mild though sub-clinical depressive symptoms may be a reflection of general chronic social adversity. Such widespread levels of mild depressive symptoms in the general clinic population highlights the importance of promoting more holistic antenatal care that attends to not only a mother's physical and mental health needs but also social needs.

Group 3 exhibits an interesting pattern, with mild levels of depressive symptoms during pregnancy that decrease and then increasing postpartum, peaking at 12 months postpartum and then dissipating by 18 months postpartum. This pattern has also been recently found among a different cohort of South African women (Garman et al., 2019). Their study used four time points (during pregnancy, 2 weeks postpartum, 6 months postpartum, and 18 months postpartum). They found moderate levels of depressive symptoms during pregnancy and 2 weeks postpartum that rose and peaked at 6 months postpartum and dissipated by 18 months postpartum. Although they did not have a 12-month postpartum assessment as we have in our study, the general trends are the same. This pattern having been identified in another study gives us more confidence that the pattern exists on a population level and is not an artifact of the data; however, further research on depression trajectories in similar samples would help in determining if this pattern exists among similar populations.

In our study, none of the measured variables predicted the pattern found in Group 3. In Garman et al. (2019), they found that both HIV status and unemployment predicted this group trajectory. However, in our study we could not test the association between HIV status and group trajectory because no HIV positive women appeared in Group 5, precluding the use of the HIV status variable in the multinomial multivariate regression model, thus, we do not know whether HIV status is a predictor of this type of trajectory in our sample. Furthermore, in our study unemployment was included in the SES composite score, which was not predictive of this depression trajectory group. A sensitivity analysis was conducted with just unemployment as a predictor of group trajectory and we did not find a significant association (data not shown). It is also possible that there are unmeasured variables that could predict this pattern including traumatic birth experiences, postpartum stressors, difficulties coping with new stresses associated with motherhood, or hormonal differences. Future research should investigate what postpartum events and changes may influences depressive symptom trajectory patterns such as this.

Both Groups 2 and 4 display opposing transient patterns of depressive symptoms; in Group 2 depressive symptoms are mild during pregnancy and increase postpartum whereas in Group 4 depressive symptoms are moderate during pregnancy and decrease to minimal levels during postpartum. Both of these trajectory types have been seen in previous studies (Baron et al., 2017, Christensen et al., 2011, Garman et al., 2019, Vänskä et al., 2011). Among the group with depressive symptoms that increase postpartum we do not know if beyond 18 months postpartum these symptoms become chronic and require intervention or if they dissipate. Future research should investigate depression trajectories beyond 18 months postpartum.

### 4.1 Limitations and strengths

There are several limitations to this study. First, there were only a few factors that significantly predicted group membership. It is likely that there are several unmeasured variables that could differentiate these groups such as birth experiences and early events postpartum, hormonal factors, family and personal histories, and genetic factors. Additionally, due to modeling limitations, we were not able to determine how psychosocial factors change, including postpartum stressful life events and postpartum exposure to trauma, and how such changes may influence depression over time. Ideally, the predictor variables would be modeled simultaneously in the group-based trajectory model. We were unable to accomplish this due to instability in the model when the predictors were entered. Furthermore, in our multinomial multivariate regression analyses, several of our groups had small sample sizes and may have contributed bias in the effect sizes (AOR). Furthermore, because of the small sample sizes in several of the groups, we may have been underpowered to detect some predictors of trajectory group. Thus, although we are confident in the significance of these predictors, the true magnitude of the effect of these predictors on group classification is unclear.

Further limitations include several differences between those included in these analyses and the full population-based sample. Those living in extreme poverty were more likely to complete more EPDS assessments, and thus, be included in this sample. Reassuringly, however, depressive symptoms at baseline were not associated with completion rates. Additionally, community and HIV status were not included in the multinomial multivariate regression because no participants from Mbekweni or participants who were HIV positive were in Group 5 and thus these variables could not be included in the regression model. TC Newman and Mbekweni are different in a number of ways that are highlighted in Table 1; however, these communities are also different in a number of cultural, linguistic, and historical ways that were not captured by these variables. Finally, the generalizability of this study may be limited. This study was conducted in a peri‑urban part of South Africa, and results may not generalize to rural areas of South Africa or to other low and middle-income country settings.

Nonetheless, there are several strengths of this study. A major strength of this study is that it is one of the first to look at longitudinal depression trajectories among pregnant and postpartum women in sub-Saharan Africa. Thus, this study helps to address a large gap in the perinatal depression trajectory literature, which tends to focus on high income, well-resourced countries, with some exceptions (Carter et al., 2019). Another strength of this study is the large sample size (*N* = 831). This is one of the largest samples used for perinatal trajectory analyses, which has allowed us to identify clinically relevant depression trajectory patterns that other studies may have been underpowered to find. Finally, this study recruited a population-based clinic sample, which adds to the generalizability of these results to similar peri‑urban areas, representing a large strength of this study.

### 4.2 Future research

Future research should investigate how the changes in stressful life events, intimate partner violence, and trauma exposure across time may impact longitudinal depression symptoms. Perinatal depression interventions should also target those with co-occurring psychosocial issues. For women with persistently high levels of depressive symptoms, we suggest that an integrated approach to dealing with perinatal depression, intimate partner violence, stressful life events, and tobacco use may be beneficial within this population. Furthermore, given the high levels of exposure to violence and trauma in this and other similar settings and the rich literature linking maternal exposure to violence and child health outcomes, future research should investigate maternal trauma/anxiety symptom trajectories across pregnancy and postpartum.

Antenatal care provides an important opportunity for the identification, referral, treatment, and management of women because the majority of South African women present to clinical care at this time. Given the widespread levels of mild depressive symptoms in the general clinic population, the promotion of more holistic antenatal care that attends to not only a mother's physical and mental health needs but also social needs, is urgently needed. Strategies for the implementation of such holistic antenatal care that can attend to transient depressive symptoms are needed particularly in resource-limited settings (Kagee et al., 2013, Price et al., 2017). Furthermore, future research needs to identify strategies for identification, referral, and treatment during the postpartum period.

## 5 Conclusions

This is one of the first studies to investigate depressive symptom trajectories among pregnant and postpartum women living in sub-Saharan Africa. Five distinct trajectory patterns were found with 82.9% of the sample in the trajectory group with mild levels of depressive symptoms with a slight decrease postpartum. Interventions to treat women with severe chronic depressive symptoms with co-occurring psychosocial issues are urgently needed.

## CRediT authorship contribution statement

**Jennifer A. Pellowski:** Formal analysis, Writing - original draft. **Angela M. Bengtson:** Formal analysis, Writing - review & editing. **Whitney Barnett:** Writing - review & editing. **Kira DiClemente:** Writing - review & editing. **Nastassja Koen:** Writing - review & editing. **Heather J. Zar:** Conceptualization, Funding acquisition, Project administration, Supervision, Writing - review & editing. **Dan J. Stein:** Conceptualization, Funding acquisition, Project administration, Supervision, Writing - review & editing.

## Declaration of Competing Interest

None

## Appendix Supplementary materials

Image, application 1
